# Interleukin‐6 levels can be used to estimate cardiovascular and all‐cause mortality risk in dialysis patients: A meta‐analysis and a systematic review

**DOI:** 10.1002/iid3.818

**Published:** 2023-04-26

**Authors:** Zeyu Chen, Yan Wang

**Affiliations:** ^1^ Department of Cardiology The First People's Hospital of Ziyang Ziyang China; ^2^ Department of Nephrology The First People's Hospital of Ziyang Ziyang China

**Keywords:** all‐cause mortality, cardiovascular‐related mortality, dialysis, interleukin‐6, meta‐analysis

## Abstract

**Background:**

Although previous studies have explored the correlation of interleukin (IL)−6 with mortality risk in dialysis patients, the findings have been conflicting. Hence, this meta‐analysis aimed to comprehensively assess the use of IL‐6 measurement for estimating cardiovascular mortality and all‐cause mortality in dialysis patients.

**Methods:**

The Embase, PubMed, Web of Science, and MEDLINE databases were searched to identify relevant studies. After screening out the eligible studies, the data were extracted.

**Results:**

Twenty‐eight eligible studies with 8370 dialysis patients were included. Pooled analyses revealed that higher IL‐6 levels were related to increased cardiovascular mortality risk (hazard ratio [HR] = 1.55, 95% confidence interval [CI]: 1.20–1.90) and all‐cause mortality risk (HR = 1.11, 95% CI: 1.05–1.17) in dialysis patients. Further subgroup analyses suggested that higher IL‐6 levels were associated with elevated cardiovascular mortality in hemodialysis patients (HR = 1.59, 95% CI: 1.36–1.81) but not in peritoneal dialysis patients (HR = 1.56, 95% CI: 0.46–2.67). Moreover, sensitivity analyses indicated that the results were robust. Egger's test revealed potential publication bias among studies exploring the correlation of IL‐6 levels with cardiovascular mortality (*p* = .004) and all‐cause mortality (*p* < .001); however, publication bias was not observed when using Begg's test (both *p* > .05).

**Conclusions:**

This meta‐analysis reveals that higher IL‐6 levels could indicate higher risks of cardiovascular mortality and all‐cause mortality in dialysis patients. These findings suggest that monitoring IL‐6 cytokine may help to enhance dialysis management and improve the general prognosis of patients.

## Graphical abstract

This meta‐analysis indicates that higher interleukin‐6 level could estimate the risk of cardiovascular mortality and all‐cause mortality in dialysis patients, indicating its monitoring may help dialysis management for improving the general prognosis

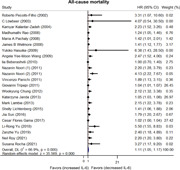

## 1 INTRODUCTION

Dialysis, which is one option for kidney replacement therapy, is commonly applied among patients with end‐stage kidney disease or acute kidney injury.^1^ Most patients require life‐long maintenance dialysis and this number is increasing globally.^2^ Unfortunately, dialysis patients present with a high mortality rate due to multiple reasons.^3^, ^4^ Cardiovascular system‐related issues are the most common causes of mortality among this group, accounting for 26.8%–40.2% of all deaths.^5^, ^6^ Hence, some novel biomarkers have been identified to estimate cardiovascular mortality in dialysis patients to improve their overall management.^7^, ^8^, ^9^

Interleukin 6 (IL‐6), a pro‐inflammatory cytokine with a 26 KDa molecular weight, is closely involved in regulating inflammation, atherosclerosis progression, and cardiovascular diseases.^10^, ^11^, ^12^, ^13^ Moreover, an increasing number of studies have investigated the correlation of IL‐6 with cardiovascular mortality in dialysis patients, but the findings have been inconsistent. For instance, several studies report that higher IL‐6 levels are related to increased cardiovascular mortality risk in dialysis patients, while other studies do not observe this correlation.^7^, ^14^, ^15^, ^16^, ^17^ Similarly, most studies report that increased IL‐6 is related to all‐cause mortality in dialysis patients, whereas some studies do not reach a similar conclusion.^18^, ^19^, ^20^, ^21^, ^22^ Therefore, it is necessary to pool these data and conduct a meta‐analysis to obtain one confirmative conclusion.

Hence, this meta‐analysis aimed to comprehensively investigate the potency of IL‐6 measurement for estimating cardiovascular mortality risk and all‐cause mortality risk in dialysis patients.

## 2 METHODS

### 2.1 Search strategy

To screen the studies that assessed the correlation of IL‐6 with cardiovascular mortality and all‐cause mortality in dialysis patients, we searched the Embase, PubMed, Web of Science, and MEDLINE databases from January 2000 to July 2022. The keywords used for searching were as follows: “interleukin‐6,” “IL‐6,” “interleukin 6,” “IL 6,” “mortality,” “death,” “survival,” “cardiovascular,” “CV,” “cardiac,” “myocardial infarction,” “dialysis,” “hemodialysis,” “HD,” “peritoneal dialysis,” and “PD.”

### 2.2 Study selection

Study screening was carried out independently by two researchers. First, the studies were searched using a predesigned information extraction form, and then the titles and abstracts were assessed. Second, the full‐text articles that satisfied the inclusion criteria were downloaded, checked, and validated. Next, studies that met the exclusion criteria were eliminated, with the reasons for exclusion were noted. Finally, the ability to extract data from the included studies was established. The reference lists of the included studies were also examined and to identify additional eligible studies. A consultation with a third investigator was conducted in cases of dispute.

Studies that met the following criteria were considered eligible: (a) study focused on the correlation of IL‐6 with cardiovascular mortality and all‐cause mortality; (b) patients received dialysis (hemodialysis or peritoneal dialysis); (c) patients were over 18 years old; (d) study had extractable and analyzable data; and (e) had at least 1 year of follow‐up. Studies were excluded based on the following criteria: (a) non‐English study; (b) systematic review, meta‐analysis, case study, case report, or animal study; and (c) duplicated study.

### 2.3 Data extraction

The data extraction was completed independently by two researchers, and in the event of a dispute, a consultation with a third researcher was performed. The extracted data included author name, publication year, sample size, patient age, dialysis type, cardiovascular mortality,^7^, ^14^, ^15^, ^16^, ^17^, ^23^, ^24^, ^25^, ^26^ and all‐cause mortality.^15^, ^17^, ^18^, ^19^, ^20^, ^21^, ^22^, ^23^, ^27^, ^28^, ^29^, ^30^, ^31^, ^32^, ^33^, ^34^, ^35^, ^36^, ^37^, ^38^, ^39^, ^40^ In addition, the hazard ratio (HR), as well as the 95% confidence intervals (CI), of IL‐6 level with cardiovascular mortality and all‐cause mortality were also collected.

### 2.4 Statistics

Data analysis and figure construction were completed using Stata (v.14.0, StataCorp). The methodological index for nonrandomized studies tool was used for quality assessment of the included studies, which involved eight dimensions and has a score ranging from 0 to 16.^41^ The Cochrane *χ* ^2^ test was used for heterogeneity analysis: the fixed effects model was used when the heterogeneity was not statistically significant (*I* ^2^ ≤ 50.0% and *p* ≥ .05), whereas the random effects model was used in cases of significant heterogeneity (*I* ^2^ > 50.0% and *p* < .05).^42^ Sensitivity analysis was carried out to assess the robustness of the data and to identify sources of heterogeneity: the statistical analyses were performed after excluding one study at a time and then the results were compared with the previous analyses. Egger's test and Begg's test were conducted to evaluate publication bias, and *p* < .05 for either test indicated potential publication bias.

## 3 RESULTS

### 3.1 Study screening and selection

**Figure 1:**
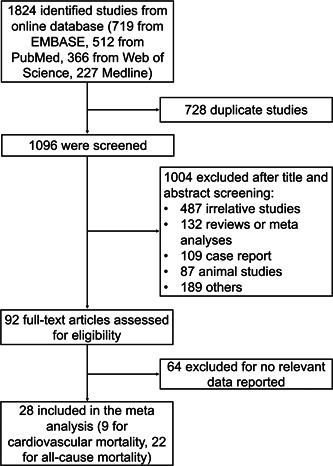
Study screening and selection process.

Initially, 1824 studies were retrieved from the online databases. Then, 728 duplicate studies were excluded. Subsequently, 1096 studies were screened, among which 1004 studies were excluded after title and abstract screening. Then, 92 full‐text articles were assessed for eligibility, among which 64 articles were excluded due to the absence of relevant data. Finally, 28 studies with 8370 patients were included in this meta‐analysis (Figure 1).

### 3.2 Characteristics of the included studies

The key information of the included studies is listed in Table 1. Subsequently, a quality assessment was conducted on these included studies (Table 2). The total quality assessment score of the included studies ranged from 12 to 14, indicating a high quality of the included studies.

**Table 1:** Details of the included studies.

No.	Author	Publication year	Continent	Sample size, n	Age (years), mean ± SD	Dialysis type	Outcomes
1	Roberto Pecoits‐Filho^27^	2002	Europe	173	53.0 ± 1.0	Hemodialysis or peritoneal dialysis	All‐cause mortality
2	C Löwbeer^18^	2003	Europe	115	52.0 ± 1.0	Hemodialysis or peritoneal dialysis	All‐cause mortality
3	Kamyar Kalantar‐Zadeh^28^	2004	America	378	54.5 ± 14.7	Hemodialysis	All‐cause mortality
4	Hirokazu Honda^7^	2006	Europe	176	54.0 ± 12.0	Hemodialysis or peritoneal dialysis	Cardiovascular mortality
5	Rulan S Parekh^14^	2008	America	1041	57.9 ± 15.0	Hemodialysis or peritoneal dialysis	Cardiovascular mortality
6	Madhumathi Rao^29^	2008	America	182	62.2 ± 12.3	Hemodialysis	All‐cause mortality
7	Maria A Pachaly^30^	2008	America	112	47.0 ± 14.0	Hemodialysis	All‐cause mortality
8	James B Wetmore^31^	2008	America	236	62.7 ± 4.3	Hemodialysis	All‐cause mortality
9	Angela Yee‐Moon Wang^15^	2009	Asia	231	55.5 ± 16.3	Peritoneal dialysis	Cardiovascular mortality, all‐cause mortality
10	Seung Hyeok Han^16^	2009	Asia	107	51.5 ± 11.2	Peritoneal dialysis	Cardiovascular mortality
11	Yukiko Hasuike^32^	2009	Asia	120	52.1 ± 0.9	Hemodialysis	All‐cause mortality
12	lia Beberashvili^19^	2010	Asia	81	64.3 ± 11.9	Hemodialysis	All‐cause mortality
13	Nazanin Noori^33^	2011	America	279	54.0 ± 15.0	Hemodialysis	All‐cause mortality
14	Vincenzo Panichi^34^	2011	America	753	60.0 ± 15.0	Hemodialysis	All‐cause mortality
15	Giovanni Tripepi^35^	2011	Europe	225	53.7 ± 16.3	Hemodialysis	All‐cause mortality
16	Wookyung Chung^36^	2012	Asia	100	53.0 ± 13.0	Hemodialysis	All‐cause mortality
17	Katarzyna Janda^23^	2013	Europe	55	53.0 ± 13.0	Peritoneal dialysis	Cardiovascular mortality, all‐cause mortality
18	Julie Calixto Lobo^24^	2013	America	45	54.6 ± 14.8	Hemodialysis	Cardiovascular mortality
19	Mark Lambie^37^	2013	America	959	55.6 ± 15.3	Peritoneal dialysis	All‐cause mortality
20	Shelly Lichtenberg^38^	2015	Asia	57	62.9 ± 16.1	Hemodialysis	All‐cause mortality
21	Jia Sun^17^	2016	Europe	543	54.4±NA	Hemodialysis or peritoneal dialysis	Cardiovascular mortality, all‐cause mortality
22	Cesar Flores Gama^20^	2017	America	153	60.5 ± 14.7	Hemodialysis	All‐cause mortality
23	Tobias Feldreich^25^	2018	Europe	228	63.0 ± 14.0	Hemodialysis	Cardiovascular mortality
24	Li‐Rong Yu^21^	2018	America	1124	61.6 ± 14.5	Hemodialysis or peritoneal dialysis	All‐cause mortality
25	Le Viet Thang^26^	2019	Asia	236	44.5 ± 14.7	Hemodialysis	Cardiovascular mortality
26	Zanzhe Yu^22^	2019	America	257	55.0 ± 14.9	Peritoneal dialysis	All‐cause mortality
27	Neil Roy^39^	2021	America	96	50.4 ± 12.8	Hemodialysis or peritoneal dialysis	All‐cause mortality
28	Susana Rocha^40^	2021	Europe	308	68.7 ± 13.6	Hemodialysis	All‐cause mortality

Abbreviation: NA, not available.

**Table 2:** Quality assessment.

Studies	A clearly stated aim	Inclusion of consecutive patients	Prospective collection of data	Endpoints appropriate to the aim of the study	Unbiased assessment of the study endpoint	Follow‐up period appropriate to the aim of the study	Loss of follow‐up < 5%	Prospective calculation of the study size	Total
Pecoits‐Filho^27^	2	1	2	2	2	2	2	0	13
Löwbeer et al.^18^	2	2	2	2	2	1	2	0	13
Kalantar‐Zadeh et al.^28^	2	1	2	2	2	2	2	0	13
Honda et al.^7^	2	1	2	2	2	2	2	0	13
Parekh et al.^14^	2	1	2	2	2	1	2	0	12
Rao et al.^29^	2	1	2	2	2	1	2	0	12
Pachaly et al.^30^	2	1	2	2	2	2	2	0	13
Wetmore et al.^31^	2	1	2	2	2	2	2	0	13
Wang et al.^15^	2	1	2	2	2	1	2	0	12
Han et al.^16^	2	1	2	2	2	2	2	0	13
Hasuike et al.^32^	2	2	2	2	2	2	2	0	14
Beberashvili et al.^19^	2	1	2	2	2	2	2	0	13
Noori et al.^33^	2	1	2	2	2	2	2	0	13
Panichi et al.^34^	2	2	2	2	2	2	2	0	14
Tripepi et al.^35^	2	1	2	2	2	1	2	0	12
Chung et al.^36^	2	1	2	2	2	1	2	0	12
Janda et al.^23^	2	2	2	2	2	2	2	0	14
Lobo et al.^24^	2	1	2	2	2	2	2	0	13
Lambie et al.^37^	2	1	2	2	2	2	2	0	13
Lichtenberg et al.^38^	2	1	2	2	2	2	2	0	13
Sun et al.^17^	2	2	2	2	2	2	2	0	14
Gama et al.^20^	2	1	2	2	2	1	2	0	12
Feldreich et al.^25^	2	1	2	2	2	2	2	0	13
L‐R Yu et al.^21^	2	2	2	2	2	2	2	0	14
Thang et al.^26^	2	1	2	2	2	2	2	0	13
Z Yu et al.^22^	2	2	2	2	2	2	2	0	14
Roy et al.^39^	2	1	2	2	2	1	2	0	12
Rocha et al.^40^	2	2	2	2	2	1	2	0	13

### 3.3 Correlation of IL‐6 with cardiovascular mortality

**Figure 2:**
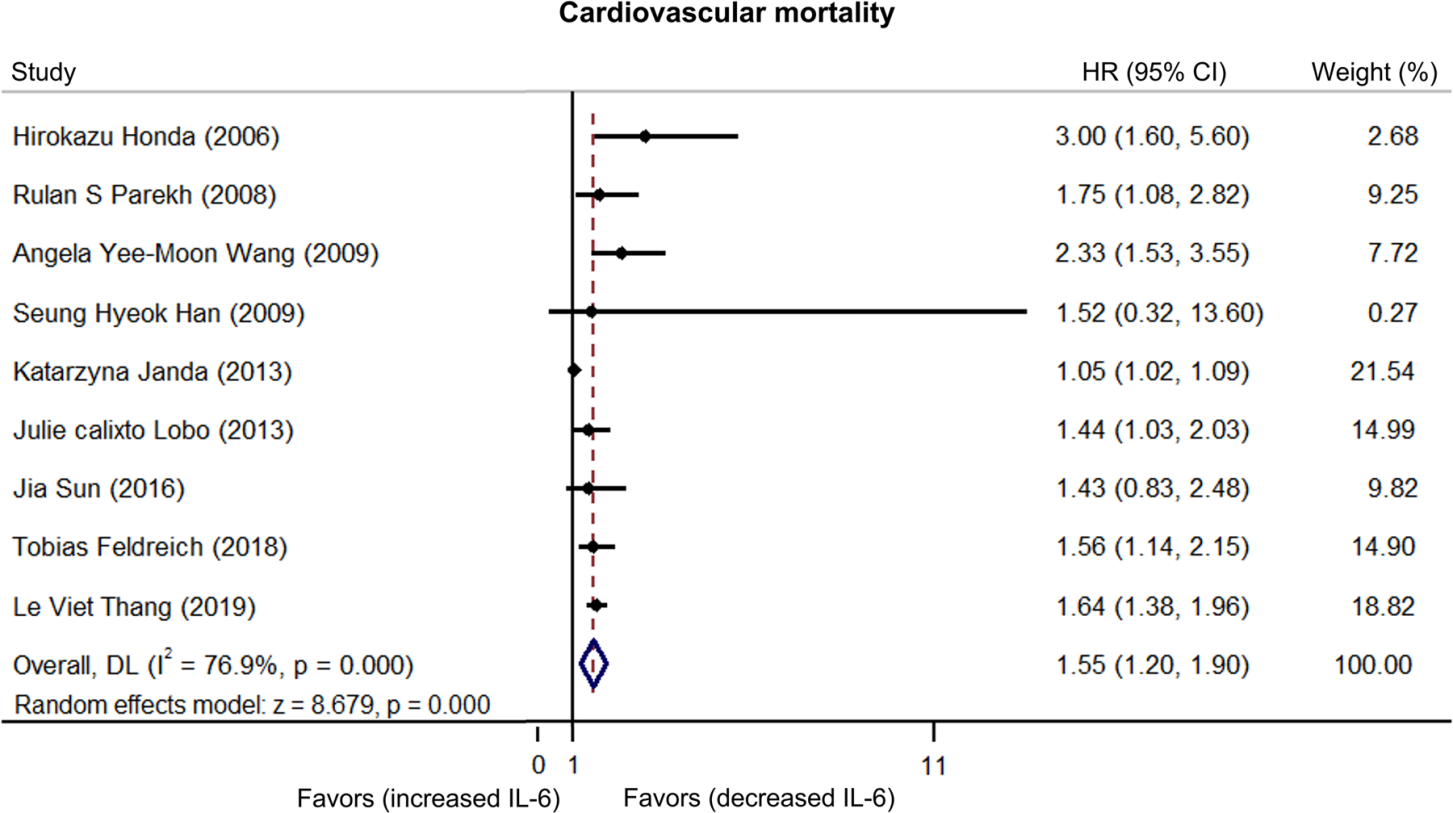
Higher interleukin‐6 (IL‐6) related to increased cardiovascular mortality risk. If the finally calculated hazard ratio (HR) value and its corresponding 95% confidence interval (CI) located in the side of “favors (increased IL‐6),” then it favored that increased IL‐6 was correlated with lower cardiovascular mortality. On the contrary, if the finally calculated HR value and its corresponding 95% CI located in the side of “favors (decreased IL‐6),” it favored that decreased IL‐6 was correlated with lower cardiovascular mortality.

**Figure 3:**
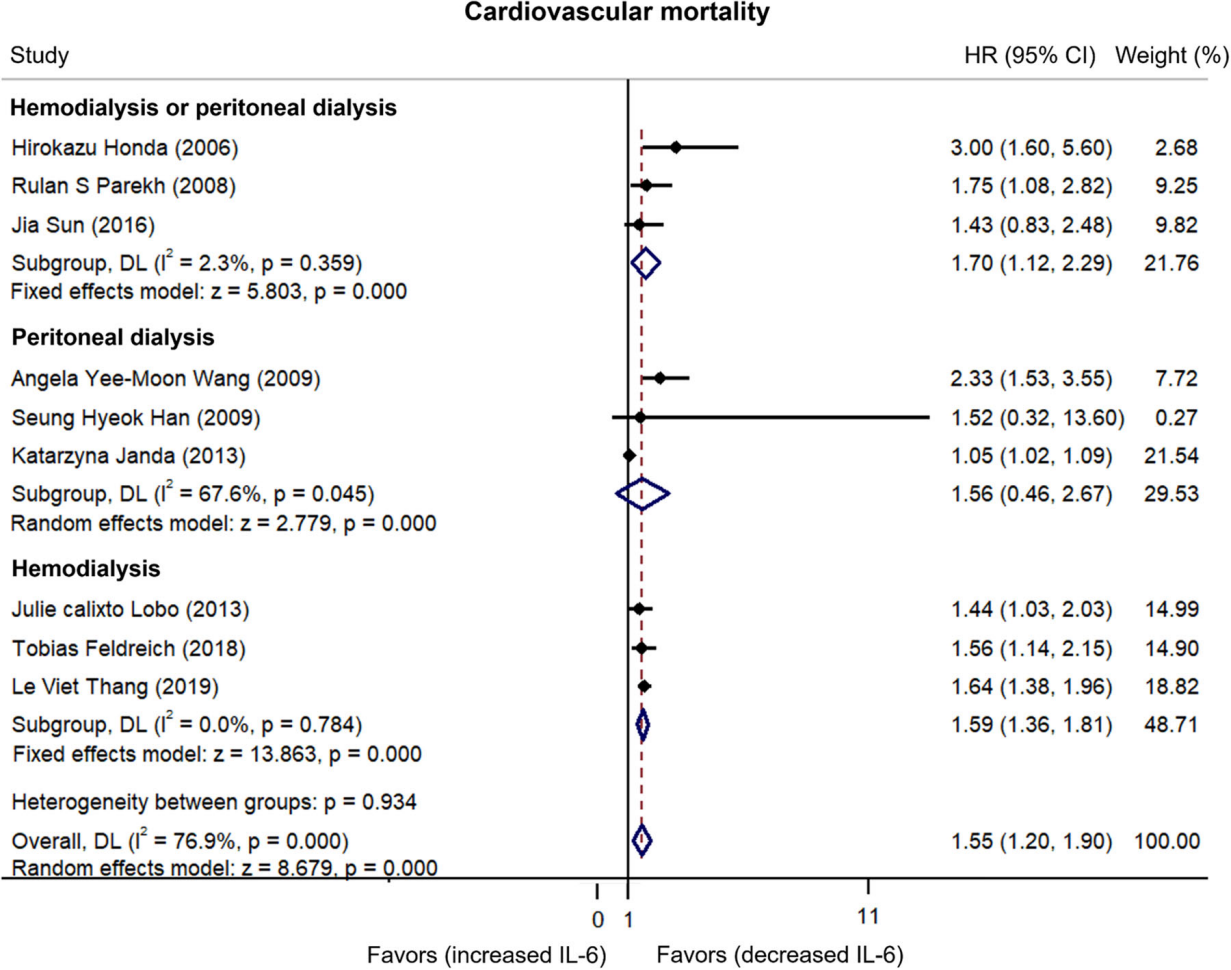
Subgroup analyses of cardiovascular mortality. If the finally calculated hazard ratio (HR) value and its corresponding 95% confidence interval (CI) located in the side of “favors (increased IL‐6),” then it favored that increased interleukin‐6 (IL‐6) was correlated with lower cardiovascular mortality. On the contrary, if the finally calculated HR value and its corresponding 95% CI located in the side of “favors (decreased IL‐6),” it favored that decreased IL‐6 was correlated with lower cardiovascular mortality.

There was heterogeneity among published articles regarding the relationship between IL‐6 and cardiovascular mortality in dialysis patients (*I* ^2^ = 76.9%).^7^, ^14^, ^15^, ^16^, ^17^, ^23^, ^24^, ^25^, ^26^ Furthermore, the pooled analysis showed that higher IL‐6 levels were related to increased cardiovascular mortality risk in dialysis patients (HR: 1.55, 95% CI: 1.20–1.90) (Figure 2). Subgroup analyses stratified by different dialysis modalities showed that IL‐6 could predict cardiovascular mortality risk in hemodialysis or peritoneal dialysis patients (HR: 1.70, 95% CI: 1.12–2.29) and hemodialysis patients (HR: 1.59, 95% CI: 1.36–1.81) but not in peritoneal dialysis patients (HR: 1.56, 95% CI: 0.46–2.67) (Figure 3).

### 3.4 Correlation of IL‐6 with all‐cause mortality

**Figure 4:**
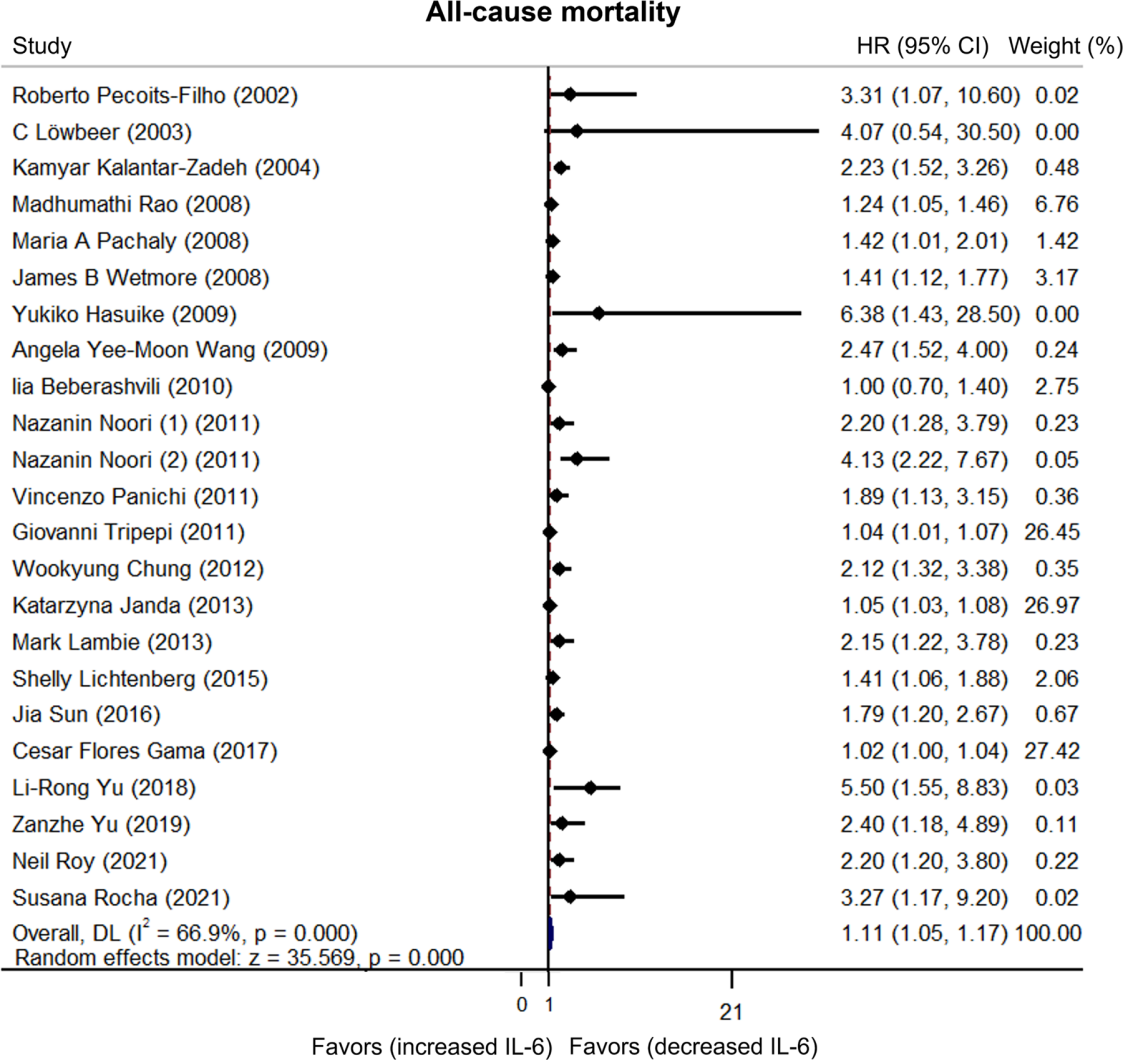
Higher interleukin‐6 (IL‐6) linked with increased all‐cause mortality risk. If the finally calculated hazard ratio (HR) value and its corresponding 95% confidence interval (CI) located in the side of “favors (increased IL‐6),” then it favored that increased IL‐6 was correlated with lower all‐cause mortality risk. On the contrary, if the finally calculated HR value and its corresponding 95% CI located in the side of “favors (decreased IL‐6),” it favored that decreased IL‐6 was correlated with lower all‐cause mortality risk.

**Figure 5:**
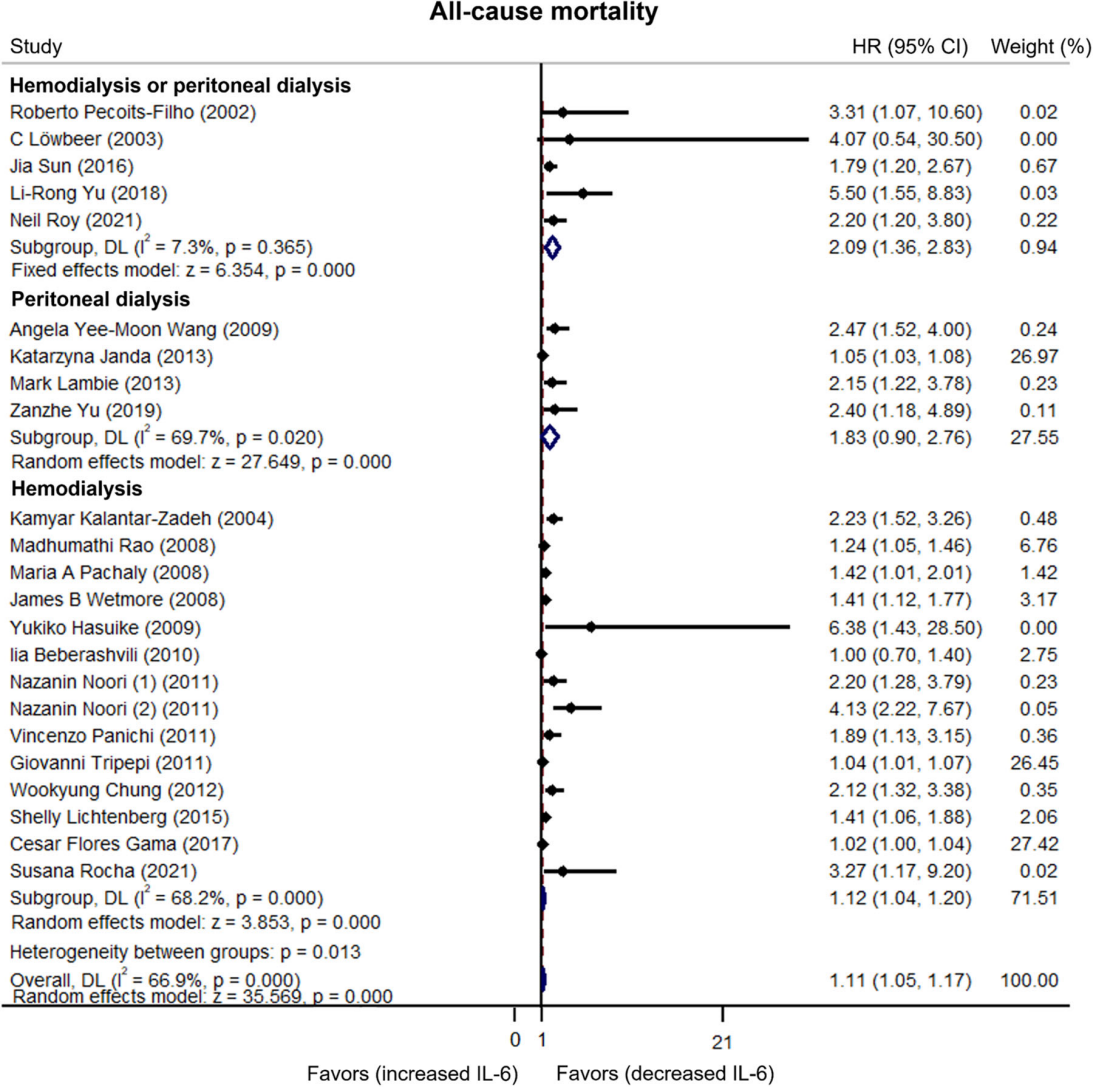
Subgroup analyses of all‐cause mortality. If the finally calculated hazard ratio (HR) value and its corresponding 95% confidence interval (CI) located in the side of “favors (increased IL‐6),” then it favored that increased interleukin‐6 (IL‐6) was correlated with lower all‐cause mortality risk. On the contrary, if the finally calculated HR value and its corresponding 95% CI located in the side of “favors (decreased IL‐6),” it favored that decreased IL‐6 was correlated with lower all‐cause mortality risk.

Heterogeneity existed among published articles regarding the relationship between IL‐6 and all‐cause mortality in dialysis patients (*I* ^2^ = 66.9%).^15^, ^17^, ^18^, ^19^, ^20^, ^21^, ^22^, ^23^, ^27^, ^28^, ^29^, ^30^, ^31^, ^32^, ^33^, ^34^, ^35^, ^36^, ^37^, ^38^, ^39^, ^40^ Moreover, pooled data showed that higher IL‐6 levels were related to increased all‐cause mortality in dialysis patients (HR: 1.11, 95% CI: 1.05–1.17) (Figure 4). Subgroup analyses stratified by different dialysis modalities revealed that increased IL‐6 levels were related to elevated all‐cause mortality risk in hemodialysis or peritoneal dialysis patients (HR: 2.09, 95% CI: 1.36–2.83) and hemodialysis patients (HR: 1.12, 95% CI: 1.04–1.20) but not in peritoneal dialysis patients (HR: 1.83, 95% CI: 0.90–2.76) (Figure 5). To clarify why IL‐6 was only correlated with cardiovascular mortality and all‐cause mortality risk in hemodialysis patients but not in peritoneal dialysis patients, we performed sensitivity analysis. The results revealed that IL‐6 predicted cardiovascular mortality (HR: 2.312, 95% CI: 1.313–3.310) and all‐cause mortality (HR: 2.330, 95% CI: 1.528–3.133) in peritoneal dialysis patients after excluding the study carried out by Janda et al.^23^ (Supporting Information: Table 1).

### 3.5 Sensitivity and publication bias

Sensitivity analyses were conducted, and it was observed that the HR did not change obviously after excluding studies individually, thus indicating the robustness of our results (Table 3). Moreover, Egger's test and Begg's test were conducted to evaluate publication bias. The results of Egger's test indicated that there was potential publication bias for cardiovascular mortality (*p* = .004) and all‐cause mortality (*p* < .001), but these findings were not significant according to Begg's test (both *p* > .05) (Table 4).

**Table 3:** Sensitivity analysis.

Omitted study	HR	95% CI	
		Lower	Upper
Cardiovascular mortality			
Honda et al.^7^	1.506	1.162	1.849
Parekh et al.^14^	1.531	1.162	1.899
Wang et al.^15^	1.476	1.133	1.819
Han et al.^16^	1.553	1.198	1.909
Janda et al.^23^	1.631	1.426	1.835
Lobo et al.^24^	1.582	1.183	1.981
Sun et al.^17^	1.570	1.191	1.949
Feldreich et al.^25^	1.555	1.166	1.944
Thang et al.^26^	1.519	1.143	1.896
All‐cause mortality			
Pecoits‐Filho^27^	1.108	1.047	1.169
Löwbeer et al.^18^	1.109	1.048	1.171
Kalantar‐Zadeh et al.^28^	1.096	1.039	1.154
Rao et al.^29^	1.096	1.035	1.158
Pachaly et al.^30^	1.103	1.042	1.164
Wetmore et al.^31^	1.094	1.035	1.154
Wang et al.^15^	1.101	1.042	1.159
Hasuike et al.^32^	1.109	1.048	1.170
Beberashvili et al.^19^	1.113	1.050	1.176
Noori et al.^33^	1.103	1.043	1.163
Noori et al.^33^	1.102	1.044	1.161
Panichi et al.^34^	1.104	1.044	1.164
Tripepi et al.^35^	1.171	1.085	1.257
Chung et al.^36^	1.101	1.042	1.161
Janda et al.^23^	1.185	1.092	1.277
Lambie et al.^37^	1.104	1.044	1.164
Lichtenberg et al.^38^	1.100	1.039	1.160
Sun et al.^17^	1.101	1.041	1.160
Gama et al.^20^	1.198	1.105	1.292
L‐R Yu et al.^21^	1.102	1.043	1.160
Z Yu et al.^22^	1.106	1.045	1.166
Roy et al.^39^	1.104	1.044	1.164
Rocha et al.^40^	1.108	1.047	1.169

Abbreviations: CI, confidence interval; HR, hazard ratio.

**Table 4:** Publication bias.

Items	Begg's test	Egger's test
Cardiovascular mortality	1.000	0.004
All‐cause mortality	0.958	<0.001

## 4 DISCUSSION

Inconsistent findings regarding the correlation of IL‐6 with cardiovascular mortality in dialysis patients have been observed.^7^, ^14^, ^15^, ^16^, ^17^ For instance, the majority of studies report that higher IL‐6 levels are related to increased cardiovascular mortality risk in dialysis patients.^7^, ^14^, ^15^ However, a small proportion of studies also disclose that higher IL‐6 levels are not related to cardiovascular mortality risk in these patients.^16^, ^17^ Therefore, this meta‐analysis was conducted, and pooled results from nine publications indicated that higher IL‐6 levels could indicate increased cardiovascular mortality risk in dialysis patients. The possible mechanism of this effect may be that (1) IL‐6 binds to its receptor IL‐6R and gp130 to further activate multiple signaling pathways (such as the Janus kinase/signal transduction and activator of transcription pathway, mitogen‐activated protein kinase pathway, and phosphatidylinositol 3‐kinase/protein kinase‐B pathway) to promote the development of atherosclerosis and chronic inflammation, which serve as key pathophysiological events for subsequent myocardial infarction and cardiovascular mortality in dialysis patients.^43^, ^44^, ^45^, ^46^, ^47^ (2) IL‐6 might also cause sudden cardiac death (SCD) through other mechanisms such as acting with direct electrophysiological effects. In detail, Inflammatory cytokines including the IL‐6 could regulate the sympathetic tone and lead to poor heart rate variability, tachycardia, and cardiac electrical instability, contributing to SCD.^48^, ^49^ Hence, increased IL‐6 levels were related to elevated cardiovascular mortality risk in dialysis patients in our pooled analysis.

Apart from cardiovascular mortality, the predictive value of IL‐6 in estimating all‐cause mortality in dialysis patients has also remained unclear. Although most studies report that increased IL‐6 is associated with higher all‐cause mortality risk in dialysis patients, two studies do not reach the same conclusion.^18^, ^19^, ^20^, ^21^, ^22^ Instead, they revealed that higher IL‐6 levels are not related to all‐cause mortality risk in dialysis patients.^18^, ^19^ In the present meta‐analysis, our pooled data showed that IL‐6 could forecast all‐cause mortality risk in dialysis patients, which could be explained as follows: (1) increased IL‐6 indicates an upregulation of inflammatory status in dialysis patients, which could be attributed to infection (including catheter‐related infection, peritonitis, bloodstream infection, and so on), metabolic syndrome, and severe pneumonia; these factors could lead to mortality in dialysis patients.^50^, ^51^, ^52^, ^53^, ^54^ (2) IL‐6 is related to cardiovascular mortality, as shown in a previous analysis, which is also a strong contributor to all‐cause mortality. Therefore, higher IL‐6 levels were associated with elevated all‐cause mortality in dialysis patients in our pooled analysis.

The mortality rate may differ in dialysis patients with different dialysis modalities. Several studies have reported a higher all‐cause mortality risk in hemodialysis patients than in peritoneal dialysis patients.^6^, ^55^ However, another study reports that dialysis modality is not associated with the mortality rate in patients with heart failure.^56^ One meta‐analysis reports a similar mortality rate between patients undergoing hemodialysis and patients undergoing peritoneal dialysis.^57^ To determine the potency of IL‐6 measurement in patients who received different dialysis modalities, subgroup analyses were performed. The findings indicated that IL‐6 could estimate both cardiovascular risk and all‐cause mortality risk in hemodialysis patients and mixed type dialysis patients but not in peritoneal dialysis patients (IL‐6 was a nonsignificant predictor of mortality risk in peritoneal dialysis patients). These interesting findings may be explained as follows. The study carried out by Janda et al.^23^ weighed heavily in the subgroup, which may inhibit IL‐6 from predicting cardiovascular mortality and all‐cause mortality in peritoneal dialysis patients; therefore, further studies with a larger sample size could be carried out to verify this finding. Apart from that, another reason for the discriminatory finding regarding the correlation of IL‐6 with the mortality between hemodialysis and peritoneal dialysis patients was that: patients with hemodialysis might undergo higher IL‐6 levels compared with those patients receiving the peritoneal dialysis according to the previous study,^58^ which implicated that intrinsic IL‐6 was of more dysregulated in hemodialysis patients compared with the peritoneal dialysis patients. It might result in more cardiovascular‐related death events and a stronger correlation (IL‐6 with cardiovascular mortality) in hemodialysis patients.

In our study, Egger's test revealed potential publication bias, which could be explained by the fact that we extracted data from Cox's regression model analyses in these published articles, and researchers might only report significant results rather than nonsignificant results. Therefore, publication bias affected our study and served as one of the major limitations. Other inherent limitations of meta‐analyses also affected the current study, such as the existence of heterogeneity, thereby limiting the generalizability of our findings. Additionally, most included studies in the current meta‐analysis were observational in nature; thus, some confounding factors might still exist and the actual risk could be underestimated.

In conclusion, our pooled data from this meta‐analysis indicate that higher IL‐6 levels could estimate the risk of cardiovascular mortality and all‐cause mortality in dialysis patients. These findings indicate that monitoring IL‐6 may be helpful for enhancing dialysis management and improving general prognosis of patients.

## AUTHOR CONTRIBUTIONS

**Zeyu Chen**: Conceptualization (lead); supervision (lead); formal analysis (equal); writing – review and editing (equal). **Yan Wang**: Methodology (lead); formal analysis (lead); writing – original draft (lead); writing – review and editing (equal).

## CONFLICT OF INTEREST STATEMENT

The authors declare no conflict of interest.

## Supporting information

Supporting Information.

## Data Availability

The data sets generated and/or analyzed during the current study are available from the corresponding author on reasonable request.
