# Supplementary material for: Interleukin‐6 levels can be used to estimate cardiovascular and all‐cause mortality risk in dialysis patients: A meta‐analysis and a systematic review
Source: Immun Inflamm Dis. 2023 Apr 26;11(4):e818. doi: 10.1002/iid3.818 (PMC10132186; doi:10.1002/iid3.818)
Supplement: Supplementary file 1 — Supporting Information. [file IID3-11-e818-s001.docx]

**Supplementary Table 1.** Sensitivity analysis for peritoneal dialysis subgroup.

| Omitted study | HR | 95% CI | |
| --- | --- | --- | --- |
|  |  | Lower | Upper |
| **Cardiovascular mortality** |  |  |  |
| Angela Yee-Moon Wang (2009) | 1.050 | 0.015 | 1.085 |
| Seung Hyeok Han (2009) | 1.586 | 0.349 | 2.824 |
| Katarzyna Janda (2013) | 2.312 | 1.313 | 3.310 |
| **All-cause mortality** |  |  |  |
| Angela Yee-Moon Wang (2009) | 1.585 | 0.648 | 2.522 |
| Katarzyna Janda (2013) | 2.330 | 1.528 | 3.133 |
| Mark Lambie (2013) | 1.770 | 0.632 | 2.909 |
| Zanzhe Yu (2019) | 1.736 | 0.704 | 2.767 |

HR, hazard ratio; CI, confidence interval.
